# Induced migration of endothelial cells into 3D scaffolds by chemoattractants secreted by pro-inflammatory macrophages *in situ*

**DOI:** 10.1093/rb/rbx005

**Published:** 2017-04-11

**Authors:** Xuguang Li, Yuankun Dai, Tao Shen, Changyou Gao

**Affiliations:** MOE Key Laboratory of Macromolecular Synthesis and Functionalization, Department of Polymer Science and Engineering, Zhejiang University, Hangzhou 310027, China

**Keywords:** cell migration, chemoattractants, endothelial cells, macrophages, scaffolds

## Abstract

Cell migration in scaffolds plays a crucial role in tissue regeneration, which can better mimic cell behaviors *in vivo*. In this study, a novel model has been proposed on controlling 3D cell migration in porous collagen-chitosan scaffolds with various pore structures under the stimulation of inflammatory cells to mimic the angiogenesis process. Endothelial cells (ECs) cultured atop the scaffolds in the Transwell molds which were placed into a well of a 24-well culture plate were promoted to migrate into the scaffolds by chemoattractants such as vascular endothelial growth factor (VEGF) and tumor necrosis factor-alpha (TNF-α) secreted by the pro-inflammatory macrophages incubated in the well culture plate. The phenotype of macrophages was mediated by 50 ng/ml interferon-gamma (IFN-γ) and different concentrations of lipopolysaccharide (LPS, 150–300 ng/ml). The cell migration depth had a positive correlation with LPS concentration, and thereby the TNF-α concentration. The ECs migrated easier to a deeper zone of the scaffolds prepared at − 10ºC (187 μm in pore diameter) than that at − 20ºC (108 μm in pore diameter) as well. The method provides a useful strategy to study the 3D cell migration, and is helpful to reveal the vascularization process during wound healing in the long run.

## Introduction

Cell migration and proliferation play a crucial role in a variety of physiological and pathological processes ranging from wound healing [1], revascularization [2], cartilage regeneration [3]. The migration of cells can be promoted under the stimuli of biochemical and biophysical signals such as mechanical property of matrix [4] and peptides [5] and growth factors in an immobilzied state and a free state [6], which are known as mechanotaxis, haptotaxis and chemotaxis, respectively [7].

Cell migration can occur two-dimensionally (2D) and three-dimensionally (3D). The investigation of cell migration *in vitro* is predominantly carried out on a planar surface, revealing the influence of stiffness [8], topology [9] and chemical structure [10] of the matrixes. Although the 2D migration results are instructive to guide the design of biomaterials for some practical applications, the 3D migration can better mimic the cell behaviors during the process of tissue regeneration *in vivo*. Indeed, there are significant differences in terms of cell adhesion and migration behaviors between the 2D and 3D environments [11]. Thus, it is of great interest and importance to study the 3D cell migration in biomaterials to provide evidences for the real processes of tissue regeneration.

Recently, the natural inflammatory response of implanted biomaterials *in vivo* has been paid much attention. In particular, the stimulation of inflammatory cells such as different phenotypes of macrophages may influence on the angiogenesis process, which is the usual case when a scaffold is implanted *in vivo* [12]. Macrophages are divided into two phenotypes: the pro-inflammatory phenotype (classically activated M_1_ phenotype) and the anti-inflammatory phenotype (alternatively activated M_2_ phenotype). When the macrophages interact with intracellular microorganisms such as viruses and bacteria, or the active stimulators including interferon-gamma (IFN-γ) and lipopolysaccharide (LPS), they are polarized to the classical M_1_ phenotype [13] with a round shape [14] in cell culture, which can produce plenty of pro-inflammatory cytokines, tumor necrosis factor-alpha (TNF-α), Interleukin (IL)-1β, reactive oxygen species (ROS), and nitric oxide (NO) [15]. When the macrophages interact with extracellular fungi and parasites, or the active stimulators including IL-4, IL-10 and IL-13, they are polarized to the alternative M_2_ phenotype [13] with a fibroblast shape [14] in cell culture, which can express a high level of anti-inflammatory cytokines, platelet-derived growth factor-BB (PDGF-BB), transforming growth factor-β (TGF-β) and matrix metalloprotease-9 (MMP-9) [15]. Some studies report that decrease of the M_1_/M_2_ ratio is beneficial for the vascularization of implanted biomaterials due to the promotion of growth factors released by the M_2_ macrophages [16], whereas other studies show that increase of the M_1_/M_2_ ratio can promote vascularization on account of the expression of potent angiogenic stimulators by M_1_ macrophages [17]. Therefore, the function of pro-inflammatory macrophages during the angiogenic process remains controversial.

In tissue regeneration, improvement of the biological accuracy of *in vitro* models is essential and significant. The models employed *in vitro* to understand cell migration for tissue repair can be sub-divided into three categories: (i) cell exclusion assay, (ii) chemotactic assay and (iii) matrix invasion assay [18]. The cell exclusion assay is an ideal model for adherent cell locomotion analysis, including scratch-induced wounds [19], stopper-based assay [20] and defined spatial inclusion assay [21]. As the name means, this method requires removing or excluding some cells in an area of the culture dish to observe the cell migration process, and thereby to mimic the wound healing. Chemotaxis is the motivation of cell migration in a specific direction in response to a gradient created by soluble attractants. Some *in vitro* models have been proposed to simulate the chemotactic attraction, for example, Transwell chamber assay [22], horizontal capillary assay [23] and microfluidic chambers [24]. During wound healing, cells show the directional migration toward the site of wounds under the mediation of several chemoattractants such as cell growth factors and cytokines. However, both the cell exclusion assays and the chemotactic assays described above are applied to the two-dimensional cell migration which could not reflect the real cell behaviors *in vivo*. Recent studies have highlighted the matrix invasion assay as the best method to mimic the environment *in vivo*. Classically, two types of biomaterials have been utilized for cell invasion: hydrogels and scaffolds. Hydrogels such as collagen [25] and fibrin [26] possess cell-specific binding sites to promote cell adhesion, but are difficult to control in terms of architecture and mechanics. Preformed scaffolds have pre-determined architecture, but cell infiltration is more challenging. To overcome this problem, cell growth factors have been incorporated into scaffolds to promote the invasion and assembly of cells [27]. However, very few attempts report the matrix invasion combined with the co-cultured cells to explore the cell–cell interaction during the regeneration process.

**Scheme 1:**
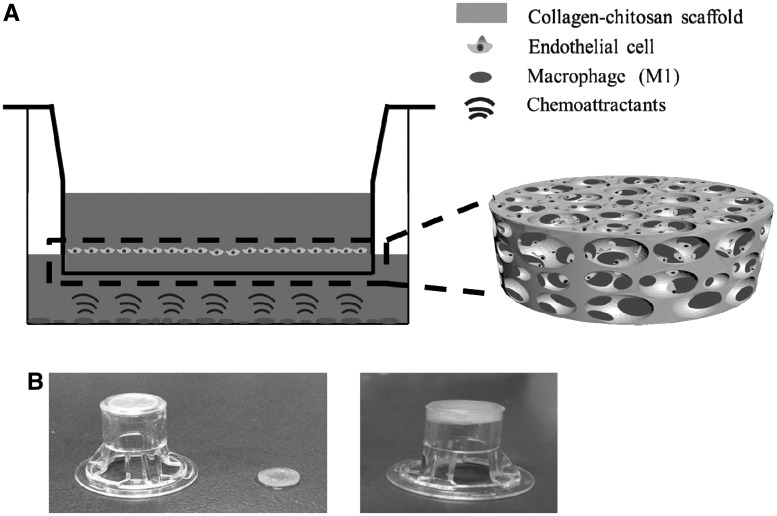
(A) schematic illustration to show the model of 3D cell migration. The pro-inflammatory macrophages with M_1_ phenotype are seeded on the bottom of a culture well. They secret chemical signals (here TNF-α and VEGF) to induce the migration of endothelial cells being seeded atop the collagen-chitosan scaffold, which is placed inside the Transwell mold. (B) (left) the Transwell mold, and (right) the Transwell mold loaded with an actual collagen-chitosan scaffold.

In this study, the Transwell molds are combined with a collagen–chitosan (C–C) scaffold, which is made of natural materials with high biostability and good biocompatibility, and has been used for dermis regeneration *in vivo* [28]. Furthermore, the C–C scaffold has pretty good angiogenesis without significant immuno-effect, and thus the auto-immuno-influence can be ruled out. Endothelial cells (ECs) are cultured atop the C–C scaffolds, which are promoted to migrate into the 3D scaffolds by biological cues such as VEGF and TNF-α secreted by the pro-inflammatory macrophages being cultured on the well of a culture plate (Scheme 1). The C–C scaffolds prepared at different temperature and thereby different pore size are used to evaluate their influence on cell migration as well. In this way, it is able to better mimic the migration behaviors of ECs during the wound healing process.

## Materials and methods

### Materials

Acetic acid solution, glutaraldehyde solution, tert-butanol and ethanol were purchased from Sinopharm Chemical Reagent Co., Ltd (China). The following materials were used as received: chitosan (M_η_ 250 kDa, deacetylation degree 85%, Haidebei, China), 2-mercaptoethanol (Sigma-Aldrich, Germany), Transwell molds (Corning, USA), 24 well culture plates (Corning, USA), Sprague–Dawley rats (120 g, Zhejiang Academy of Medical Science), Dulbecco’s modified eagle medium (DMEM, Gibco), penicillin (CSPC PHARMA.) and streptomycin (Lukang PHARMA.), fetal bovine serum (FBS, Sijiqing Inc., Hangzhou, China), recombinant human macrophage colony stimulating factor (MCSF, Peprotech, catalog no.300-25), interferon-gamma (IFN-γ, PROSPEC, catalog no.cyt-358), lipopolysaccharide (LPS, Escherichia coli O111:B4, catalog no.L2630), bovine serum albumin (BSA, AMResco, CAS# 9048-46-8), fluorescein isothiocyanate (FITC)-conjugated mouse antibodies against CD68 (Abcam, catalog no.BYK-0271 R), phycoerythrin (PE)-conjugated mouse antibodies against CC chemokine receptor type-7 (CCR7, Affymetrix eBioscience, catalog no.12-1971), 4’,6-diamidino-2-phenylindole (DAPI, Sigma), rhodamine- phalloidin (Invitrogen), and Triton-X100 (Sigma-Aldrich, CAS# 9002-93-1).

### Preparation and characterization of collagen-chitosan scaffolds

The collagen–chitosan (C–C) scaffold was prepared by a freezing-drying method [29]. Collagen type I was isolated from bovine tendon by method of pepsin digestion and acid swollen, and sufficiently dialyzed against water to remove salts and other small molecules before lyophilization. A 0.5% (w/v) collagen solution was prepared in 3% (w/v) acetic acid solution, which was mixed with a 0.5% (w/v) chitosan/acetic acid solution with a ratio of 9:1. 15 min later, the mixture was crosslinked with 0.25% (w/v) glutaraldehyde solution for 4 h at 37 ºC. The mixture was frozen for 24 h at −10ºC or −20ºC. The porous C–C scaffold was obtained by freeze-drying for 24 h. The disk-shape scaffold of 1 mm in diameter was prepared and immersed into 75% (v/v) ethanol for sterilization. Finally, the C−C scaffold was washed with sterile phosphate buffered saline (PBS, pH 7.35) to rinse off alcohol before cell culture.

The Hitachi S-4800 scanning electron microscope (SEM) was used to characterize the morphology of C−C scaffold at an operating voltage of 3.0 kV. The cross-sections of samples were sputtered with gold before analysis. The pore sizes were determined by measuring random 100 pores from SEM images using the ImageJ software (Sun Microsystems, Inc., National Institute of Health, USA).

Porosity was measured by a liquid displacement method. In brief, ethanol is a non-solvent for the C−C scaffold, and can easily infiltrate into the scaffold with negligible swelling or shrinkage. The scaffold was cut into a known volume (V_1_), whose weight (m_1_) was measured by a balance. After being immersed in ethanol for 1 day, the total weight of ethanol-impregnated scaffold (m_2_) was measured again. Taking into account the ethanol density (0.79 g/cm^3^), the porosity was calculated by (m_2_−m_1_)/(0.79*V_1_).

### Isolation and polarization of macrophages

The macrophages (M_0_) were isolated from Sprague–Dawley rats in accordance with the ‘Guidelines for Animal Experimentation’ by the Institutional Animal Care and Use Committee, Zhejiang University. Briefly, 10 ml DMEM was injected into the enterocoelia of each rat, followed with massage for 3 min and non-disturbance for 7 min. The medium was then extracted, and the released cells were collected by centrifugation at 1000 rpm/min. Next, 3 × 10^5^ cells were incubated in a well of 24 well culture plate containing 1 ml DMEM supplemented with 100 U/ml penicillin and 100 µg/ml streptomycin, 10% fetal bovine serum and 0.05 mM 2-mercaptoethanol at 37ºC and 5% CO_2_.

Polarization was implemented by using fresh medium supplemented with 20 ng/ml MCSF and following cytokines: 50 ng/ml IFN-γ and different concentrations (150, 200, 250 and 300 ng/ml) of LPS. After polarization for 48 h, the macrophages were continuously incubated in a fresh medium with the same cytokines for the following cell migration experiments.

The expression of surface antigens of glycoprotein CD68 and CCR7 was evaluated to analyze the polarization degree of macrophages by flow cytometry (FACSCalibur, BD). In brief, 10^5^ macrophages being polarized or not were incubated in 500 μL PBS containing specific antibodies of FITC-conjugated mouse antibodies against CD68 (dilution 1:12.5) and PE-conjugated mouse antibodies against CCR7 (dilution 1:10) at 37ºC for 1 h. Corresponding isotype controls without antibody label were used as recommended by the manufacturers for comparison with each antibody. The antibody-labeled cells were pretreated with 1% BSA/PBS to prevent the non-specific adsorption, washed twice in PBS, and analyzed by using a FACSCalibur flow cytometer and the CellQuest software (BD Biosciences, Pharminogen).

Enzyme-linked immunosorbent assay (ELISA) kits from BOSTER were used to measure the secreted mouse TNF-α and VEGF from concentrated supernatant after the macrophages were polarized for 48 h, respectively. The supernatant of macrophages without polarization was used as a control. After centrifugation, the supernatant was diluted was 1:2 with 2% BSA/PBS before the measurement was performed according to the manufacturer’s protocols. Briefly, the antibody specific for TNF-α or VEGF was pre-coated onto the wells of a microplate. The standards and samples were pipetted into the wells. After removal of any unbound substances, the biotin-conjugated antibody specific for TNF-α or VEGF was added to the wells. After washing avidin-conjugated horseradish peroxidase (HRP) was added to the wells. Following a wash to remove any unbound avidin-enzyme reagent, tetramethylbenzidine solution was added to the wells, and the blue color was developed in proportion to the amount of the proteins. The color change was measured by a microplate reader (TECAN, INFINITE 200 PRO) at a wavelength of 450 nm. The concentration of TNF-α or VEGF in the samples was then determined by comparing the O.D. of the samples to a standard curve constructed at the same conditions. Data were reported in pg/ml.

To observe the morphology and identify the macrophage phenotypes, the cells were stained with CCR7 by using the antibodies described above (dilution 1:400). The nuclei of cells were stained with DAPI (dilution 1:250). The immunofluorescence images were acquired by a fluorescence microscope (OLMPUS, LX2-UCB).

### 3D migration of ECs into C–C scaffolds induced by macrophages

Human vein ECs were obtained from the Cell Bank of Typical Culture Collection of Chinese Academy of Sciences (Shanghai, China). The ECs were maintained with high-glucose DMEM supplemented with 10% fetal bovine serum (FBS), 100 U/ml penicillin and 100 μg/ml streptomycin, and were cultured at 37 °C in a 5% CO_2_ humidified environment. After the cells reached about 80% confluence (about 3 days), they were detached and serially subcultured. The ECs at passage 2 (P2) were used in this study.

In the first study to monitor the ECs migration into the C–C scaffold, the C–C scaffold prepared at −10°C was conditioned with complete DMEM for 2 h before cell seeding and insertion into the Transwell chamber. Then the scaffold-containing Transwell chamber was placed into a well of a 24 well culture plate, on the bottom of which 3 × 10^5^ macrophages with different phenotypes were pre-seeded in 500 μl medium. As a control, the culture medium containing 300 ng/ml LPS alone with and without macrophages was used to reveal the possible role of LPS on cell migration. Equal number of ECs (3 × 10^5^) suspended in 200 μl medium was then seeded atop each scaffold to determine the migration behaviors. Fresh medium was replenished twice for a total period of 5 days, and the stimulators for the macrophages were remained constant. The whole setup was incubated at 37°C and 5% CO_2_ for different time_._

In the second study to compare the migration behaviors of ECs in the C–C scaffolds prepared at −10 and −20°C, the macrophages were polarized by 50 ng/ml IFN-γ and 300 ng/ml LPS, and the culture time was varied. The migration of ECs in the scaffolds without any stimulation (LPS or IFN-γ) was conducted and used as a control. The medium was changed at days 1, 4 and 7 with constant concentrations of stimulators, whereas the medium of control samples was changed with complete DMEM in the same frequency.

The EC migration depth was monitored by confocal laser scanning microscopy (CLSM, ZEISS LSM780, Germany). Briefly, at pre-determined time intervals the cells-seeded scaffolds were washed three times with PBS. ECs were fixed for 1 h with 3.9% paraformaldehyde in PBS, followed by three washes. The ECs were permeabilized by using 0.1% Triton-X100 for 5 min at 4°C, followed by three washes, and then incubated with 1% BSA/PBS for 1 h. Finally, the ECs in the scaffolds were stained with rhodamine–phalloidin (dilution 1:400) and DAPI (dilution 1:100) at 37°C for 1 h. Series images in Z direction with a 5-μm step were taken by CLSM with an 20× objective lens and excitation wavelengths of 405 and 561 nm, respectively. The Z series images were reconstructed into a composite image by using the ZEN software. The migration pattern of ECs was recorded according to the appearance and disappearance of the two individual channels (red for cytoskeleton and blue for nuclei) under similar conditions. Averages of three values were taken for comparing the migration depth.

### Cell morphology and viability in the collagen–chitosan scaffolds

The morphology of ECs on or within the scaffold was observed by scanning electron microscopy. The cells cultured for 7 days in the C–C scaffold prepared at −10°C were fixed in 3.9% paraformaldehyde/PBS solution for 1 h, and then immersed sequentially in 25, 50, 75 and 100% ethanol solutions, each for 15 min to dehydrate. It was further immersed in tert-butanol to replace the ethanol. Finally, the sample with cells was freeze-dried for 24 h, sputter coated with gold and observed under SEM.

3-(4,5-Dimethyl-2-thiazolyl)-2,5-diphenyl-2-H-tetrazolium bromide (MTT) was used to measure the viability of ECs being cultured in the C–C scaffolds prepared at −10°C every 2 days until day 7. At the time point for viability assay, the scaffolds were incubated in 1 ml compete DMEM medium containing 100 μl 5 mg/ml MTT solution at 37°C for 4 h. After the scaffolds were cut into small pieces and placed into centrifuge tubes, 1 ml dimethyl sulphoxide (DMSO) was added to dissolve the formed formazan crystals for about 15 min. The solution was centrifuged at 1000 rpm for 5 min, and the absorbance of the supernatant, which was diluted twice, was measured at 565 nm by a microplate reader.

### Statistical analysis

All cell migration experiments were run in triplicate and results are expressed as mean ± SD (*n* = 3). Statistical analysis of data was performed by one-way analysis of variance (ANOVA). The statistical significance was set as *P* ≤ 0.05.

## Results

### Morphology of C–C scaffolds

**Figure 1:**
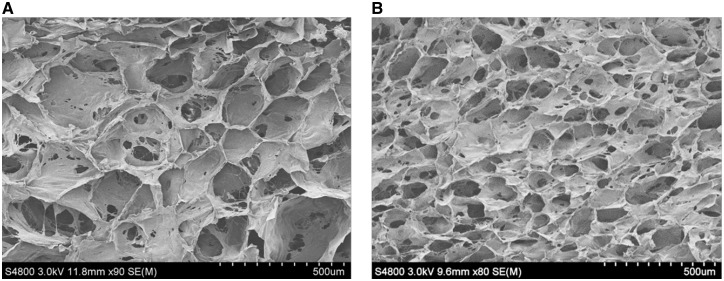
Scanning electron microscopy images of collagen–chitosan scaffolds prepared at − 10 °C (A) and (B) −20 °C, respectively

The C–C scaffolds were endowed with various pore structures relying on the temperature regimes during the freeze-drying process [30]. In this study, the C–C scaffolds were frozen at −10 and −20°C, respectively. Figure 1 shows that they had the compact and partially open pore structures regardless of the freezing temperature, but the average pore size of −10°C scaffold (186.7 ± 15.0 μm) was significantly larger than that of −20°C scaffold (108.1 ± 13.0 μm). The relatively lower freezing temperature results in a faster cooling rate, leading to smaller ice crystals which correspond to the pores after lyophilization [31]. There is no significant difference in terms of the porosity for the scaffolds obtained at −10°C (80 ± 3.6%) and −20°C (82 ± 7.7%) since it is mainly dependent on the polymer concentration rather than the freezing temperature [32]. Depending on the types of tissue to be regenerated, the scaffolds with porosity higher than 80% are usually high enough for dermis regeneration and angiogenesis [33].

### Assessment of cytocompatibility of C–C scaffolds

In order to observe the cell morphology in the scaffolds, the ECs were loaded into the C–C scaffolds prepared at −10°C. The morphology of the cells was nearly round on the surface (Supplementary Fig. S1A) and in the pore wall of the scaffolds (Supplementary Fig. S1B) with plenty pseudopodia and good adhesion with the matrix. This is apparently different from that on a planar substrate [7], suggesting the restriction and limitation effect of the 3D environment.

To investigate the cytocompatibility of the C–C scaffolds, the viability of ECs being cultured in the scaffolds was measured by MTT assay. Supplementary Figure S1C reveals that the cytoviability was not changed significantly during the 7-day culture, although a slight increasing tendency can be concluded. Nonetheless, these results suggest that the C–C scaffolds are non-toxic to the ECs.

### Characterization of polarized macrophages

**Figure 2:**
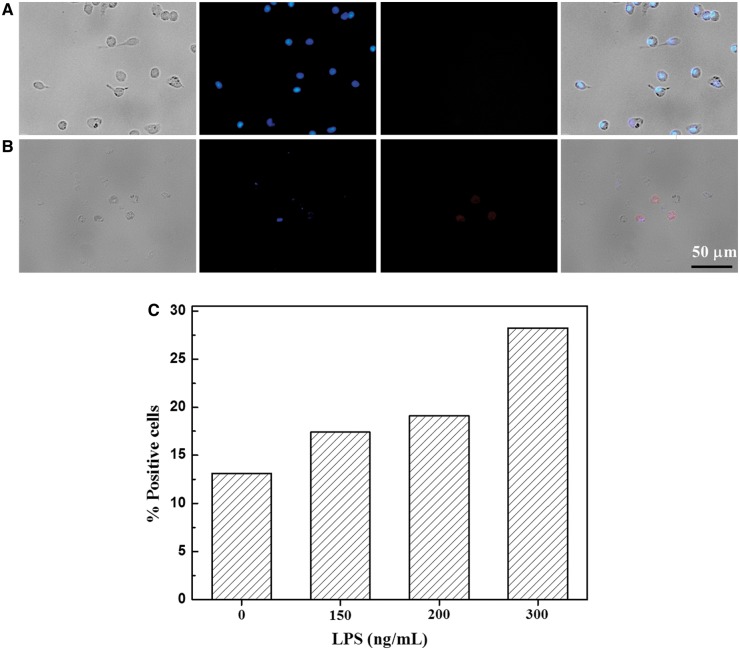
Bright field and representative CCR7 immunofluorescence images of (A) M_0_ macrophages, and (B) M_1_ phenotype macrophages being polarized by 50 ng/ml IFN-γ and 300 ng/ml LPS for 48 h. Blue: nucleus stained by DAPI; red: immunofluorescence staining of CCR7. (C) the percentage of CCR7-positive cells vs LPS concentration after the macrophages were polarized for 48 h

The macrophages isolated by intraperitoneal injection were activated by MCSF, and polarized to the pro-inflammatory macrophage phenotypes (M_1_) via specific cytokines (IFN-γ and LPS) [15]. In this study, the concentration of IFN-γ was fixed at 50 ng/ml, whereas that of the LPS was varied. The macrophage phenotypes were compared with an inactivated control phenotype (M_0_). The M_0_ macrophages had essential pseudopodia (Fig. 2A), whereas the pro-inflammatory macrophage phenotype (M_1_) showed primarily round shape (Fig. 2B).

It is known that the M_1_ macrophage up-regulates the surface marker CCR7 [15]. Immunofluorescence staining revealed that the CCR7 antibody expression (Fig. 2B, in red) was mostly on the surface of the M_1_ macrophages, which were stained with DAPI in blue. Comparatively, the expression of CCR7 was stronger on the pro-inflammatory macrophages, but little on the inactivated control phenotype (Fig. 2A). Hence, the macrophages were successfully polarized to the pro-inflammatory macrophage phenotype via the specific cytokines at the given concentrations (50 ng/ml IFN-γ and 300 ng/ml LPS).

Flow cytometric analysis was used to further quantify the macrophage phenotypes. The surface marker CD68 is strongly expressed on the inactivated control phenotype (M_0_) [34]. After the macrophages were collected from peritoneum, the fraction of CD68+ macrophages was as large as 90.2%, suggesting that most cells isolated by intraperitoneal injection were macrophages (M_0_). After the polarization CCR7 was expressed more strongly on the M_1_ macrophages than that on the M_0_ macrophages, and was enhanced along with the increase of LPS concentration (Fig. 2C).

**Figure 3:**
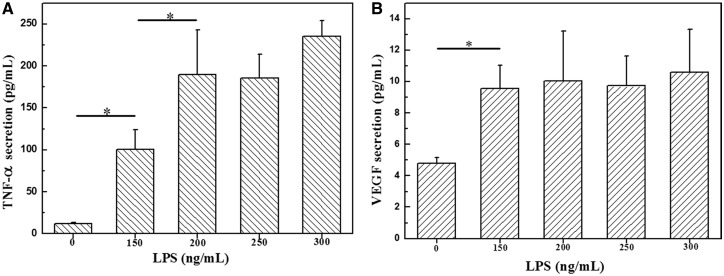
Secretion of (A) TNF-α and (B) VEGF by macrophages being polarized with different concentrations of LPS for 48 h

Since the CCR7 can also be expressed weakly by other phenotypes of macrophages such as M_2_a and M_2_c [15], the secretion of proteins by the pro-inflammatory macrophages was further testified to verify the functions of polarized macrophages. Figure 3A shows that the secretion of TNF-α by the polarized macrophages increased significantly along with the increase of LPS concentration until 200 ng/ml, and then did not change significantly. The secretion of VEGF was also significantly enhanced upon co-incubation with 150 ng/ml LPS compared with that by the M_0_ macrophages, but no significant increase of the VEGF secretion was observed with still higher concentrations of LPS (Fig. 3B). The inflammatory cytokines TNF-α secreted by the M_1_ macrophages can stimulate the ECs, upregulate vascular EC growth factor receptor-2 (VEGFR2) expression [35], and prime ECs for sprouting to promote cell migration [36]. Furthermore, VEGF is a potent inducer for migration and tube formation of ECs, and is a key mediator in the process of angiogenesis [37].

### Assessment of cell migration within 3D collagen-chitosan scaffolds

**Figure 4:**
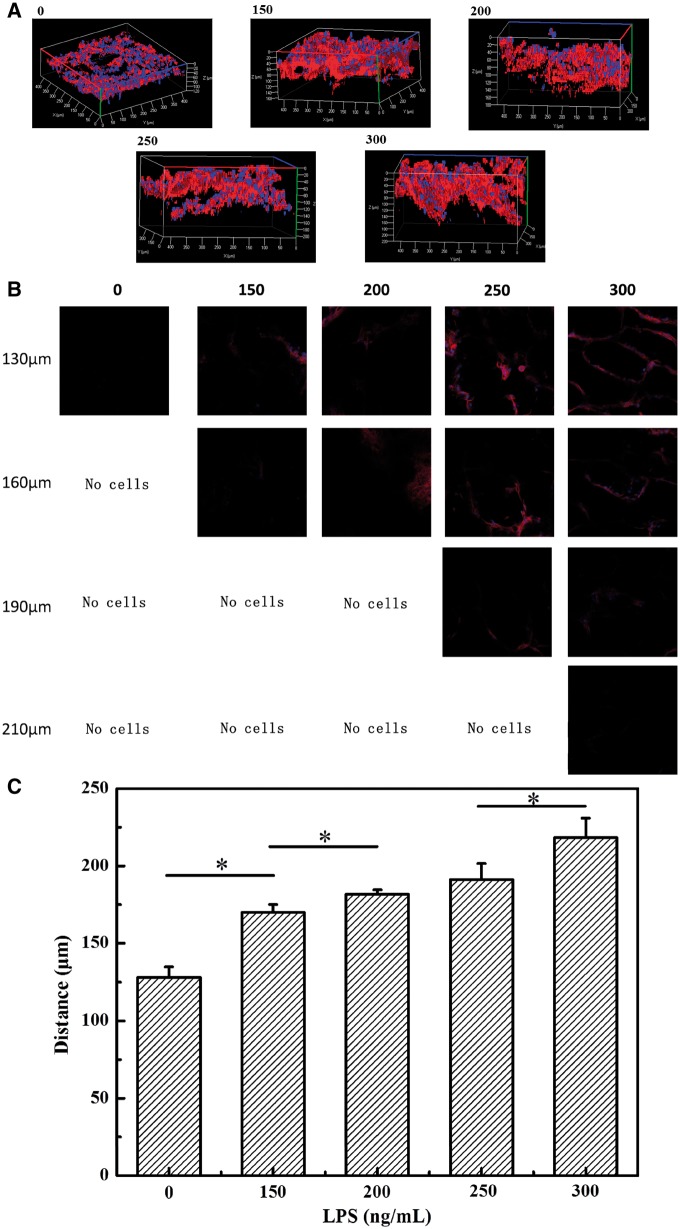
(A) 3D-reconstructed confocal images of ECs after being cultured for 5 days atop collagen-chitosan scaffolds prepared at − 10 °C, under which macrophages being polarized with different concentrations of LPS were seeded. (B) cross sectional images in Z direction with a 30 μm distance were taken by CLSM with an 20× objective lens and excitation wavelengths of 405 and 561 nm. Blue: nucleus stained by DAPI; red: cytoskeleton stained with rhodamine-phalloidin. (C) migration depth of ECs into the scaffold as a function of concentration of LPS used to polarize the macrophages

It is crucial to assess the effect of chemical signals on the cell migration within a 3D matrix. In this study, the migration behaviors of ECs were studied by using the model shown in Scheme 1. The concentrations of chemoattractants could be controlled by addition different concentrations of LPS as shown in Figure 3. In the first experiment, the C–C scaffolds prepared at -10 °C were used, while the LPS concentrations were varied (0, 150, 200, 250 and 300 ng/ml). The migration distance from the top surface to the inside was quantified from CLSM images at day 5. The dense cell cytoskeletons in red and nuclei in blue were clearly visible (Fig. 4A and B), and the cells arranged tightly around the pores in the scaffolds (Fig. 4B). As the concentration of the LPS increased, the ECs could migrate deeper into the scaffolds (Fig. 4C). The ECs migrated the longest distance at the LPS concentration of 300 ng/ml, which is significantly longer than that at lower concentrations of LPS and the control sample. To exclude the possible influence of LPS on cell migration, the ECs were cultured in mediums with and without 300 ng/ml LPS and macrophages, respectively (Supplementary Fig. S2). Without macrophages, the migration distance of ECs was independent on the existence of LPS, demonstrating that the LPS alone is not able to effectively induce ECs migration. Comparatively, the inactivated macrophages without LPS (M_0_) could significantly induce the ECs migration, which is consistent with the results shown in Figure 3, and this effect should be attributed to the low level of VEGF and TNF-α secreted by the Mo.

**Figure 5:**
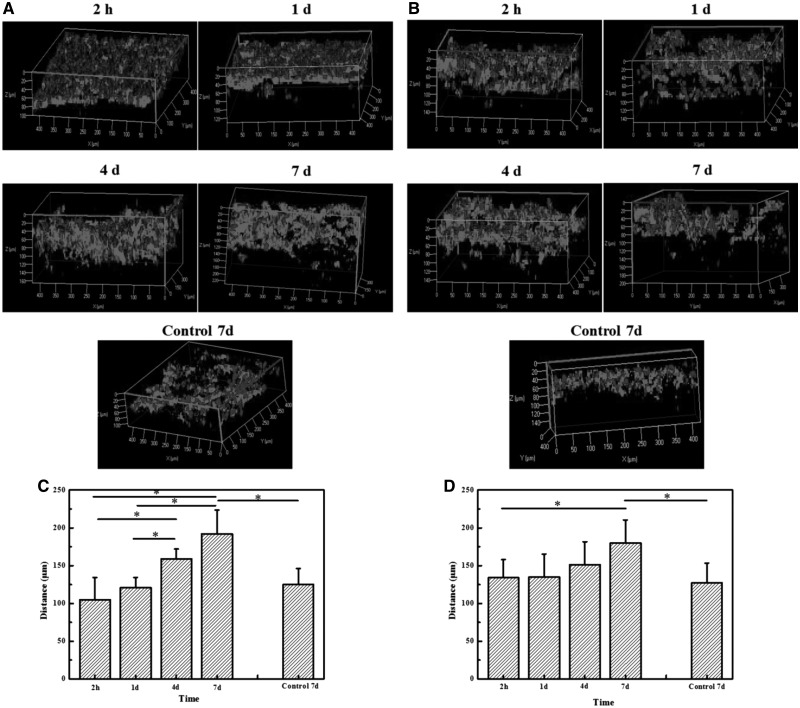
3D-reconstructed confocal images of ECs after being cultured atop collagen–chitosan scaffolds prepared at (A) −10 °C and (B) −20 °C for different time, under which macrophages being polarized with 300 ng/ml LPS were seeded, respectively. Migration depth of ECs into (C) −10 °C and (D) −20 °C scaffolds as a function of culture time, respectively. The migration of ECs into the scaffolds without macrophages was used as a control, respectively

In the second study, the concentration of LPS was fixed at 300 ng/ml, whereas the scaffolds prepared at −10°C (Fig. 5A) and −20°C (Fig. 5B) were used to compare the migration ability of ECs. The ECs migrated into the deeper zone of the scaffolds prepared at −10°C along with the prolongation of culture time, and reached about 200 μm in depth at day 7 (Fig. 5C). The apparent net migration distance reached to 87 μm. By contrast, the migration depth of control cells without macrophage seeding was significantly shorter, and was comparable with the very initial value at 2 h of the experimental cells. Unlike the gradual migration into the scaffolds prepared at −10°C, the ECs firstly reached a pretty deeper zone of the scaffolds prepared at −20°C, and then kept insignificant movement until day 7, at which the depth of migration was about 180 μm (Fig. 5D). During the process, the net distance was 46 μm. Taking into account the migration process and distance, the scaffold prepared at −10°C with larger pores allows easier migration of ECs than that prepared at −20°C with smaller pores, although the latter shows pretty good ECs infiltration too.

## Discussion

In this study, we have developed a model to mediate cell migration in 3D porous collagen–chitosan scaffolds by using inflammatory macrophages with an ability to release chemoattractants, which is able to mimic the revascularization process of the implanted scaffolds *in vivo*. Along with the increase of LPS concentration, the polarization of the macrophages is enhanced, which produces larger amount of VEGF and TNF-α, leading to the consistent deeper migration distance of ECs in the C–C scaffolds. It is known these factors can enhance the proliferation and migration of ECs [27,36], a major type of cells responsible for the formation of new blood vessels. Comparatively, the concentration of secreted VEGF at different polarized conditions is not significantly different, while that of the TNF-α does, suggesting that the TNF-α plays a more important role in the inductive migration process of ECs. Nonetheless, TNF-α and VEGF may mediate cross-talk between macrophages and ECs at sites of inflammation, resulting in an enhanced and temporally regulated burst of angiogenesis.

The microstructures of the scaffolds could also influence the cell migration behaviors to some extent. The migration of ECs in the −10°C scaffolds has a similar regime as that of the TNF-α concentration, likely conveying the positive correlation. As time goes by, the cells could migrate deeply into the scaffolds during 7 days. In particular, at day 4 the distance of cell migration into the −10°C scaffolds increased significantly, likely due to the pulse of TNF-priming ECs for rapid sprouting at 2–3 days [36]. No such gradual migration of ECs in the −20°C scaffolds was observed, suggesting that the bigger pore size of −10°C scaffold leads to better cell migration [38]. In addition to the microstructures, there are some other physical or mechanic properties that may affect 3D cell migration. For example, the stiffness of hydrogel could influence cell migration and morphology; the cells form spheroids and aggregate in the stiffer hydrogels, which restrain the cell migration ability [39]. Moreover, the scaffolds with moderate wettability improve attachment, growth and migration of cells, since their surface has preferential absorption of cell-adhesive proteins [40]. Lastly, the surface roughness of a scaffold has an important influence on protein absorption and thereby cell adhesion and migration upon implantation *in vivo* [41].

Our work highlights a novel concept on controlling 3D cell migration in porous scaffolds with various pore structures which could be applied to the tissue engineering of skin and other tissues [28]. Recently, the most studied approach to the cell migration is based on the planar 2D surfaces, which could analyze the real-time orientation and migration rate of cells. However, the 2D migration reveals better haptotatic migration, but not the cell interaction, and thereby is not able to completely mimic cell migration behaviors *in vivo*. Due to the essential difference between the 2D microenvironment and the real microenvironment *in vivo*, the 3D cell migration could better reflect the cell behaviors in the scaffolds during the processes of tissue regeneration. Based on the 3D cell migration, the cytokines and chemokines interacted with cells have been paid much attention. For example, Zhang *et al.* [42] created an *in situ* matrix environment that is conducive to MSCs with robust migration depending on the stromal cell-derived factors-1 (SDF-1) to promote cartilage self-repair. Y.H. Shen *et al.* [27] conjugated VEGF onto collagen scaffolds using N-(3-dimethylamino-propyl)-N′-ethylcarbodiimide hydrochloride chemistry, and found that the immobilized VEGF promotes the penetration and proliferation of ECs in the collagen scaffolds. However, our model is based on the cascade of cell-cell interactions to mimic the self-healing process of tissues, and demonstrates the role of inflammatory cells on the migration of ECs into the scaffolds. It can better mimic the angiogenesis process mediated by macrophages upon the scaffolds are implanted *in vivo*.

Besides of the current focus, it has been a popular topic to study the inflammatory cells-induced cell migration. For example, tumor-associated macrophages (TAMs) are correlated with poor prognosis in many human cancers. Activated macrophages promote the migration and cytoskeleton rearrangement of cancer cells, which are determined by Transwell migration assay too [43]. So far very a few attempts have combined the inflammatory cells-induced migration of somatic cells with scaffolds whose properties can be adjusted to observe the process of 3D migration. The difficulty is to measure the cell migration into the matrix in a real-time manner, and thus the model is rarely reported.

The novel models will provide a useful strategy to study the 3D cell migration, which could also be applied to other types of cells. For example, mesenchymal stem cells cultured atop the scaffolds are recruit into the materials by chemoattractants such as PDGF [44] secreted by the alternative M_2_ phenotype for the repair of a number of damaged tissues. Moreover, the model could be modified by regulating the immune response of macrophages with cell–cell interaction instead of the active molecular stimulators. The macrophages co-cultured with A549 respiratory epithelial cells, which could modulate the expression of transcription factor NF kappa B [45], are able to be activated to the M_1_ phenotype as the signal resource to induce the migration of ECs or EC clusters into scaffolds. Future work will focus on the intelligence-responsive materials with local degradable and remodeling features via cell-material interaction, which can better reveal the actual processes of tissue regeneration in biodegradable scaffolds.

## Conclusion

An original model of 3D migration was proposed in this study, which was based on M_1_ macrophages-induced migration of ECs into porous collagen-chitosan scaffolds. The ECs could migrate into a deeper zone along with the gradual enhancement of chemotaxis adjusted by the feeding concentration of LPS. Furthermore, the pore size of the scaffolds played a role in the cell migration and infiltration to some extent. The relative simplicity of the method will provide a useful strategy to study the 3D cell migration, and reveal the vascularization during wound repair process in the long run.

## Supplementary Material

Supplementary Data
