# Supplementary material for: Induced migration of endothelial cells into 3D scaffolds by chemoattractants secreted by pro-inflammatory macrophages in situ
Source: Regen Biomater. 2017 Apr 11;4(3):139–48. doi: 10.1093/rb/rbx005 (PMC5458538; doi:10.1093/rb/rbx005)
Supplement: Supplementary Data [file rbx005_Supp.doc]

**Supporting Information for**

Induced migration of endothelial cells into 3D scaffolds by chemoattractants secreted by pro-inflammatory macrophages *in situ*

Xuguang Li, Yuankun Dai, Tao Shen, Changyou Gao*

MOE Key Laboratory of Macromolecular Synthesis and Functionalization, Department of Polymer Science and Engineering, Zhejiang University, Hangzhou 310027, China.

*Corresponding author.

Email: [cygao@mail.hz.zj.cn](mailto:cygao@mail.hz.zj.cn)

Fax: +86-571-87951108


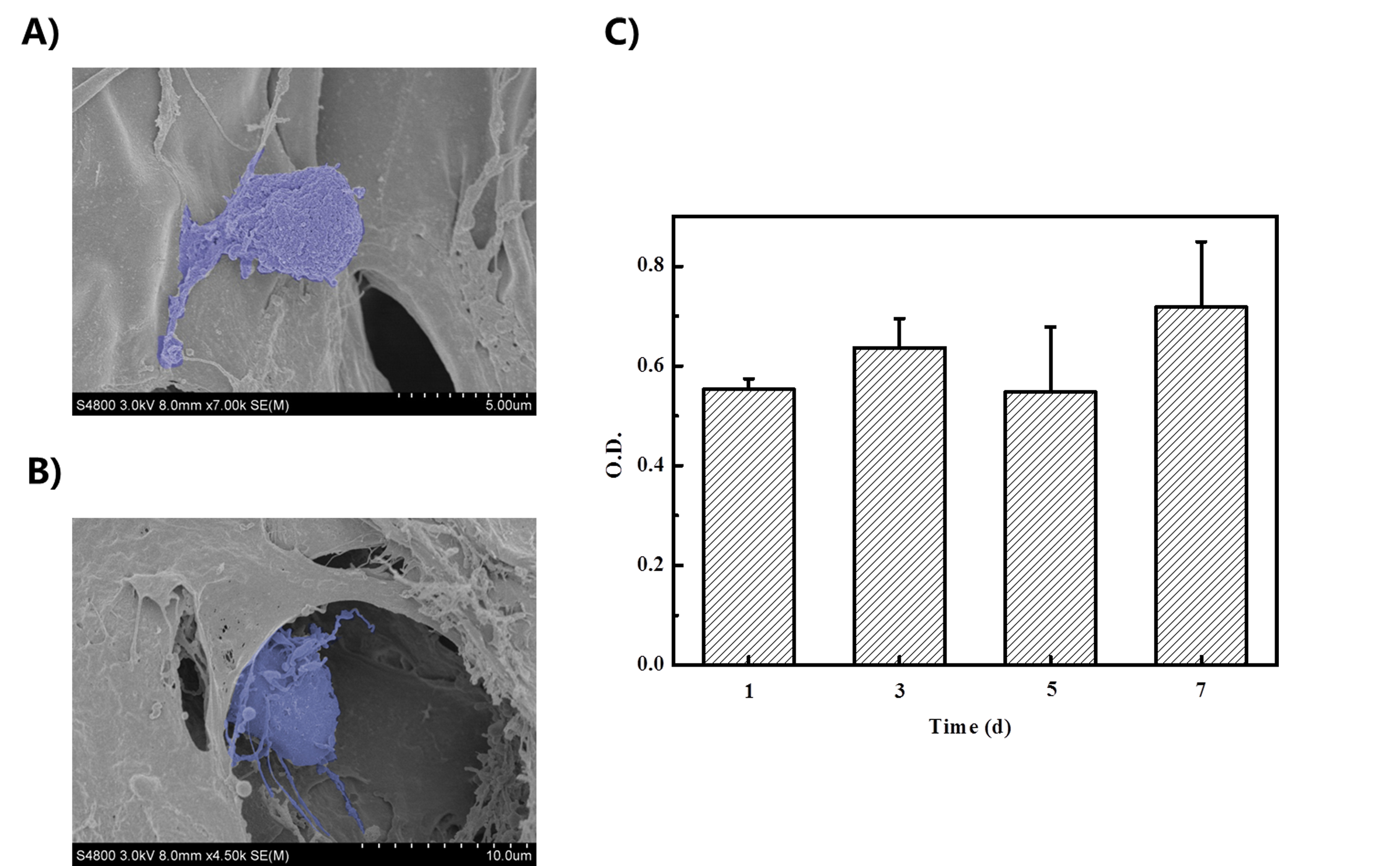


Fig. S1 Compatibility of endothelial cells in C-C scaffold prepared at -10 ^o^C. (A,B) SEM images of endothelial cells of blue color on surface (A) and inside (B) the scaffold after being cultured for 7 d. (C) Optical density (O.D.) of MTT assay of cells in the scaffolds as a function of time.


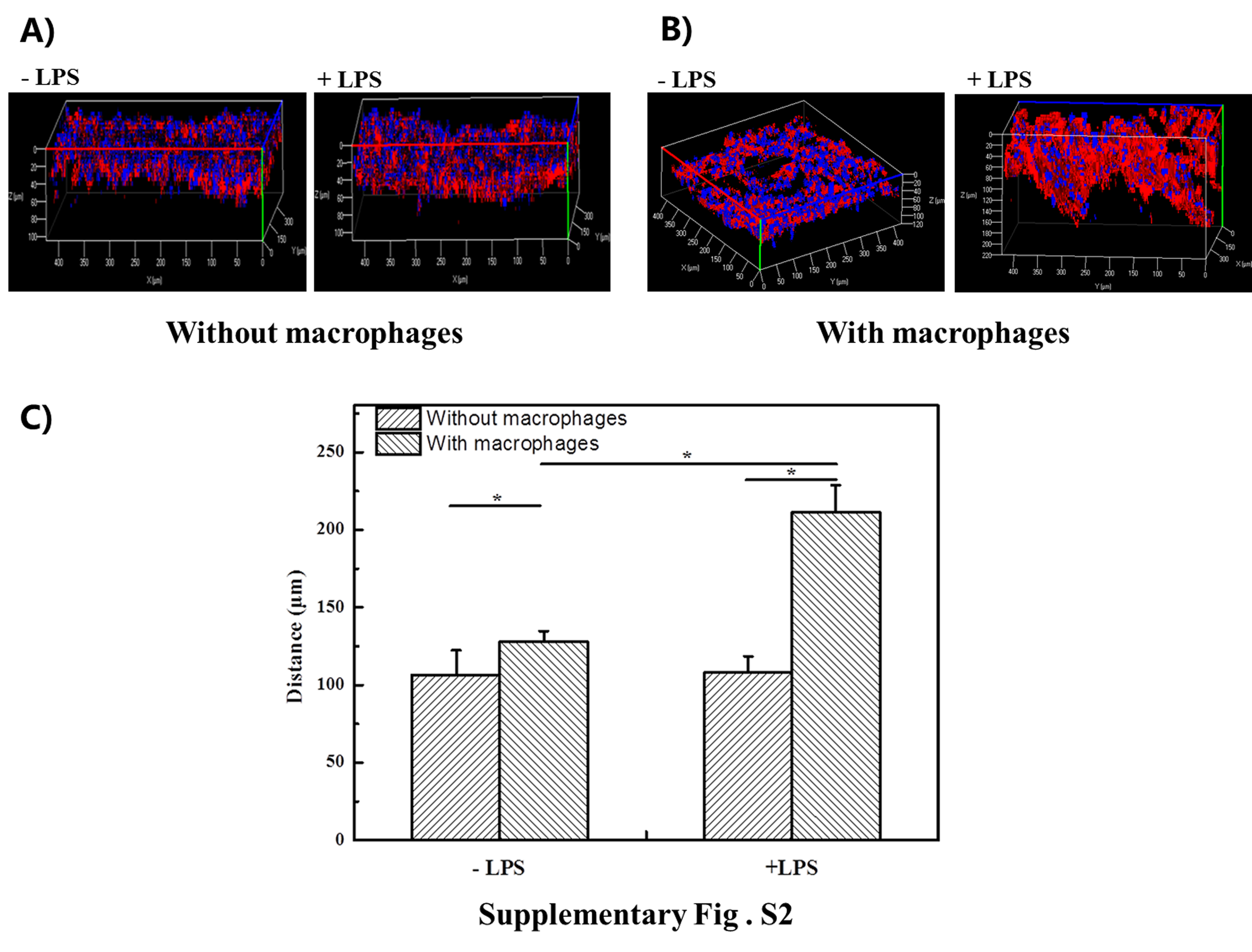


Fig. S2 3D-reconstructed confocal images of ECs after being cultured for 5 days atop collagen-chitosan scaffolds prepared at -10 ^o^C without (A) and with (B) macrophages seeded underneath. 300 ng/mL LPS was either added (+LPS) or not (-LPS) as noted in the figure. (C) Quantitative migration depth of ECs into the scaffold at different conditions as noted in the figure. * present statistical difference at *p*<0.05 level.
